# Supplementary figures and images for: Damage sensing mediated by serine proteases Hayan and Persephone for Toll pathway activation in apoptosis-deficient flies
Source: PLoS Genet. 2023 Jun 15;19(6):e1010761. doi: 10.1371/journal.pgen.1010761 (PMC10270351; doi:10.1371/journal.pgen.1010761)

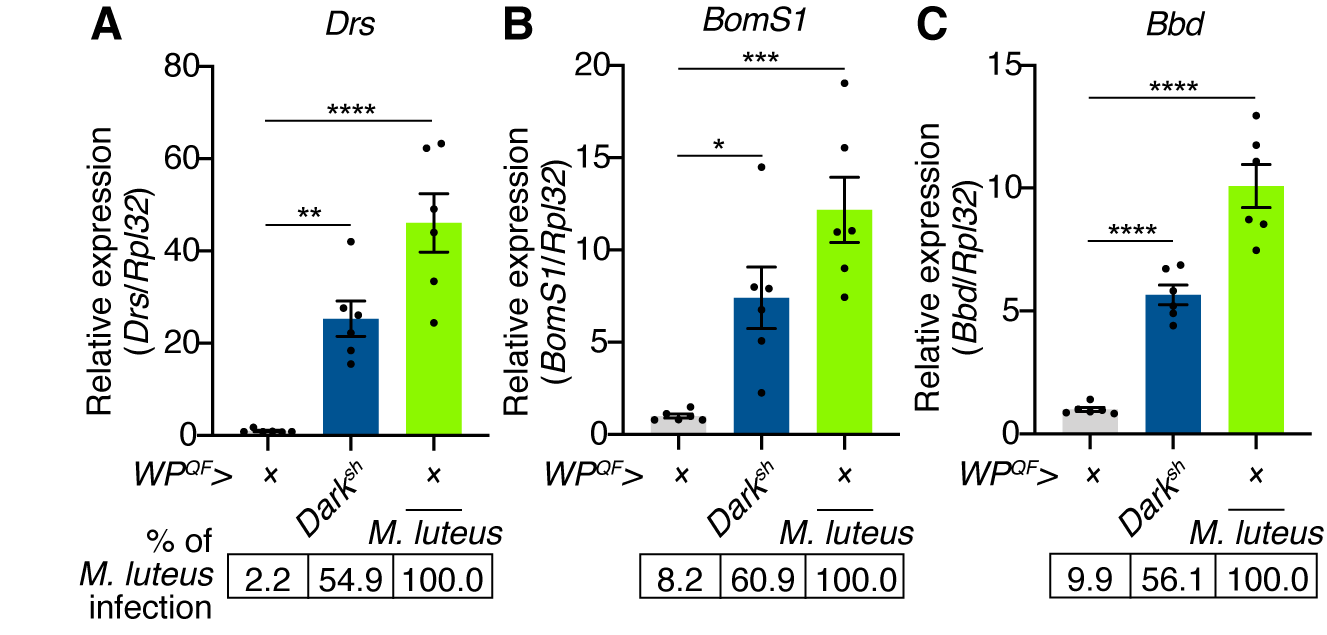

Supplement: S1 Fig — (A–C) Quantitative RT-PCR of Drs (A), BomS1 (B), and Bbd (C) in the whole body of control male flies (WPQF>+), apoptosis-deficient male flies (WPQF>Darksh), and male flies with septic injury with M. luteus (WPQF>+, M. luteus) raised on a standard diet. Expression levels of Toll target genes under each condition are listed in the table, with those of flies with M. luteus infection as 100%. n = 6. Data are mean with SEM. Each dot represents a replicate. Statistical analysis was performed using one-way ANOVA with Tukey’s multiple comparison test. *: P < 0.05; **: P < 0.01; ***: P < 0.001; ****: P < 0.0001. (TIF) [file pgen.1010761.s001.tif]

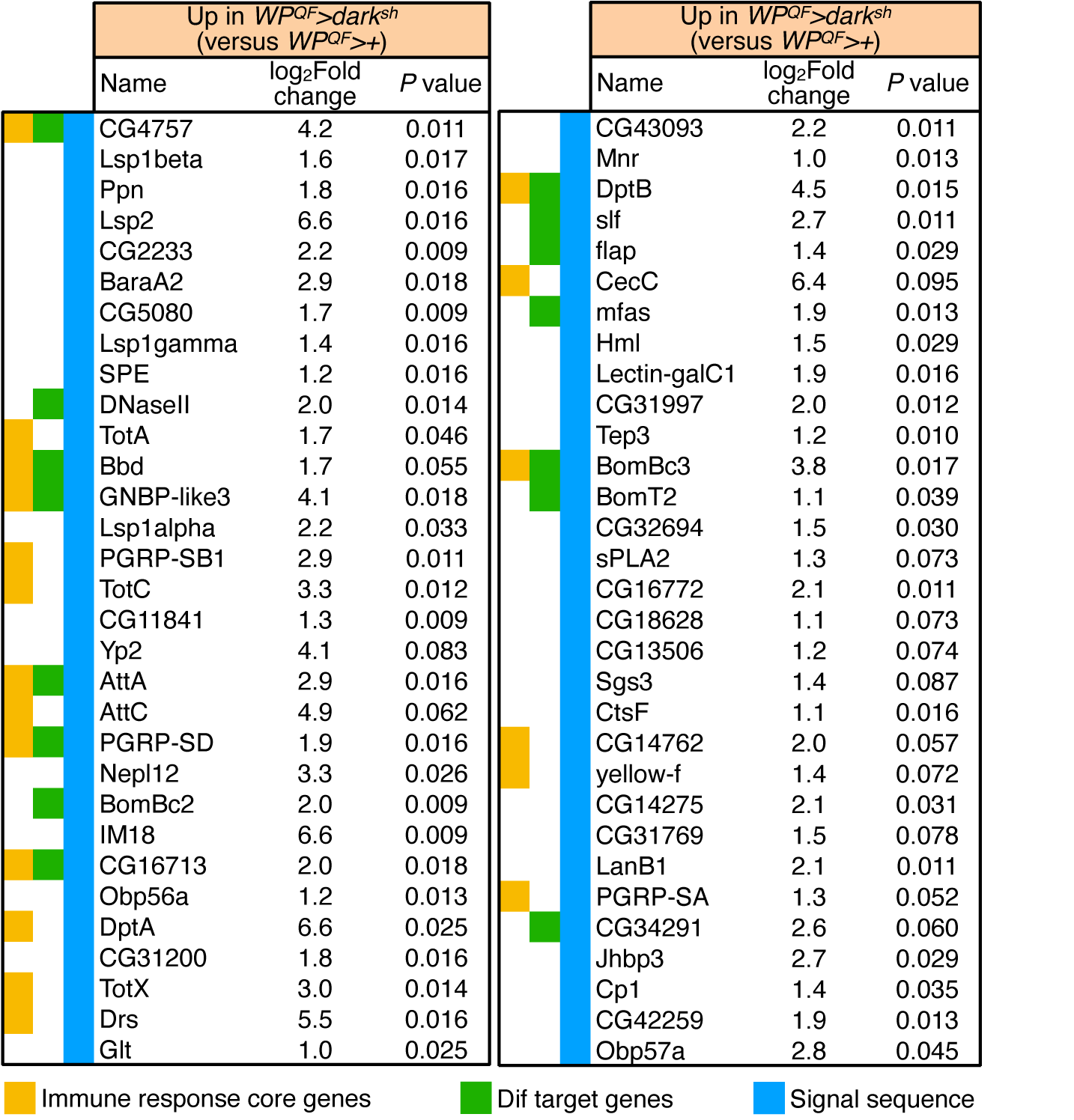

Supplement: S2 Fig — Proteomic analysis of adult hemolymph from control (WPQF>+) and apoptosis-deficient (WPQF>Darksh) male flies at 6 days after eclosion. Three biological replicates were prepared for each genotype. List of upregulated proteins (log2Fold change ≥ 1.0, adjusted P value < 0.1) having the putative signal sequence in the hemolymph of apoptosis-deficient flies compared with that of control flies. A color scale on the left side of the list shows whether the genes encoding each protein are known to be an immune responsive core gene (yellow), have the putative Dorsal-related immune factor (Dif) binding sites (green), or have the putative signal sequence (light blue). (TIF) [file pgen.1010761.s002.tif]

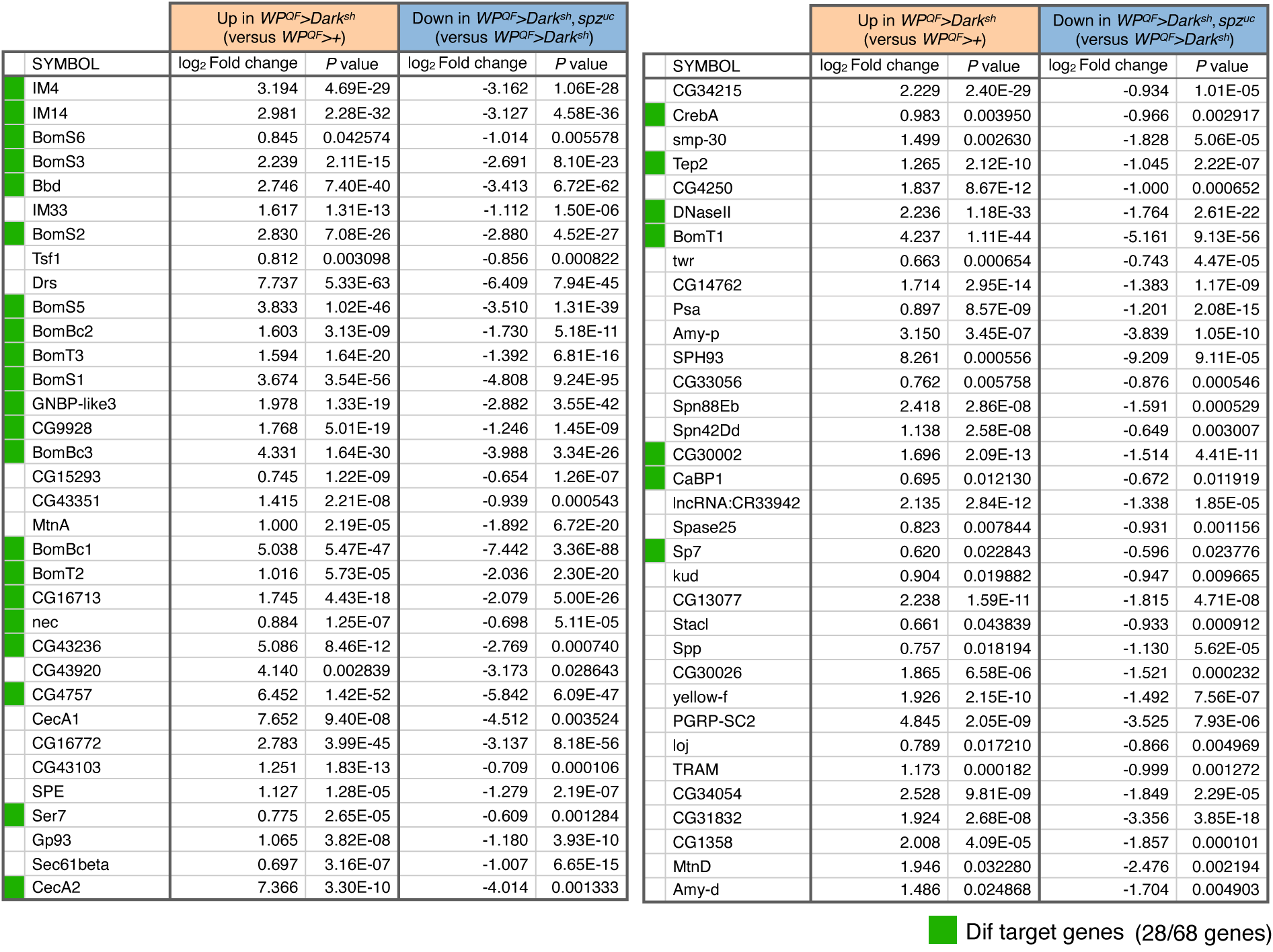

Supplement: S3 Fig — From the RNA-seq analysis, the differentially expressed genes, whose expression was upregulated in apoptosis-deficient flies (WPQF>Darksh versus WPQF>+) but downregulated in spzuc-background flies (WPQF>Darksh, spzuc versus WPQF>Darksh), were listed with Benjamini–Hochberg adjusted P value <0.05 after Wald test. i-cisTarget analysis identified 28 putative Dif target genes in the 68 genes, indicating that apoptosis deficiency induces Toll pathway activation through Spz cleavage. (TIF) [file pgen.1010761.s003.tif]

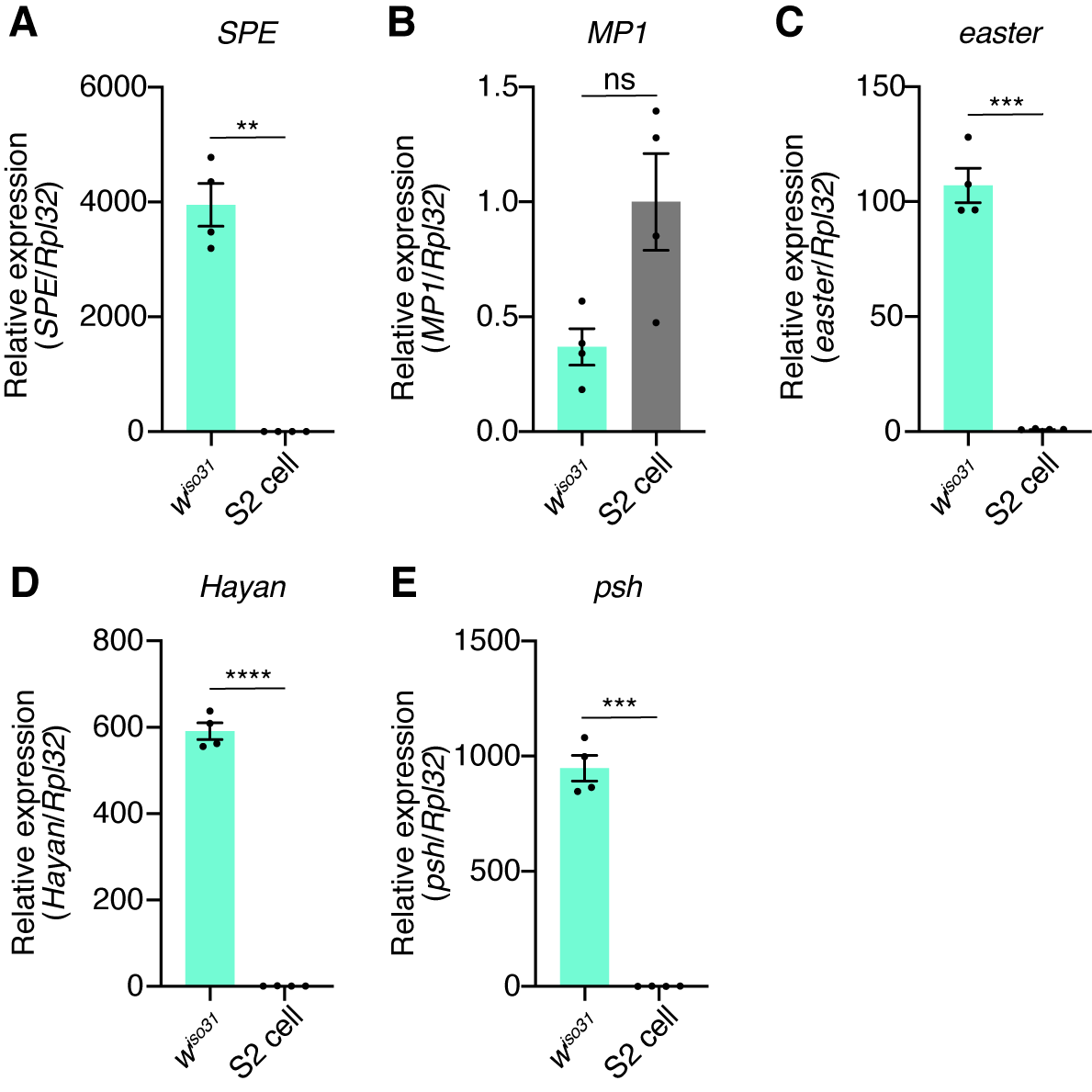

Supplement: S4 Fig — (A–E) Quantitative RT-PCR of SPE (A), MP1 (B), easter (C), Hayan (D), and psh (E) in the whole body of control (wiso31) male fly and S2 cell samples. n = 4. Data are mean with SEM. Each dot represents a replicate. Statistical analysis was performed using two-tailed Welch’s t test. ns: P > 0.05; **: P < 0.01; ***: P < 0.001; ****: P < 0.0001. (TIF) [file pgen.1010761.s004.tif]

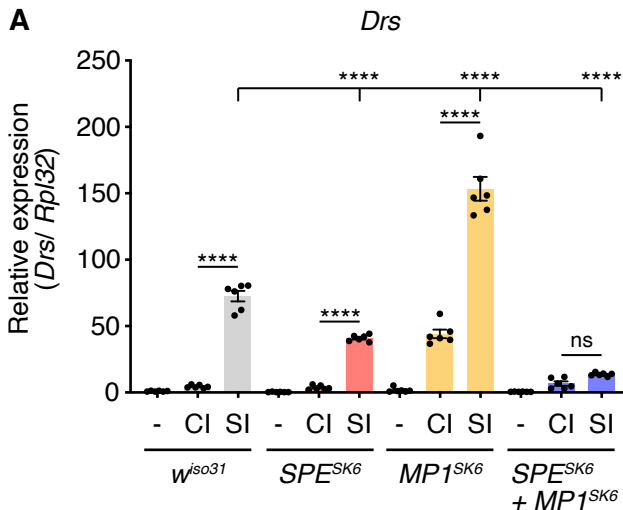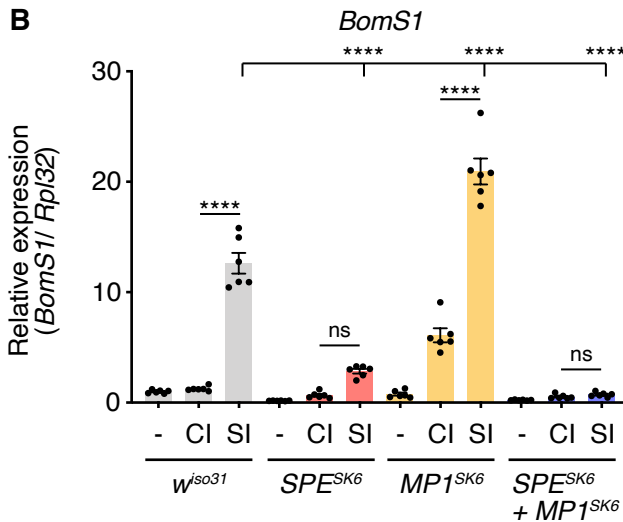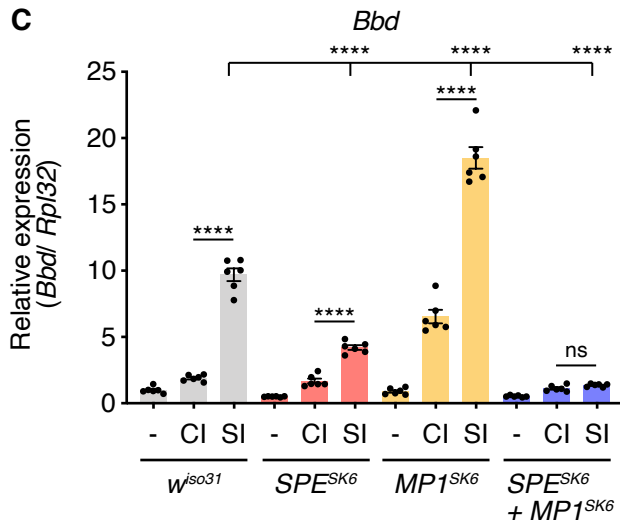

Supplement: S5 Fig — (A–C) Quantitative RT-PCR of Drs (A), BomS1 (B), and Bbd (C) in the whole body of control (wiso31) and SP mutant (SPESK6, MP1SK6, or SPESK6 + MP1SK6) male flies raised on a standard diet. Each group of flies was either untreated or treated, with either clean or septic injury. Fly samples were collected 24 hours after treatment. n = 6. Data are mean with SEM. Each dot represents a replicate. Statistical analysis was performed using one-way ANOVA with Tukey’s multiple comparison test. ns: P > 0.05; ****: P < 0.0001. (PDF) [file pgen.1010761.s005.pdf]

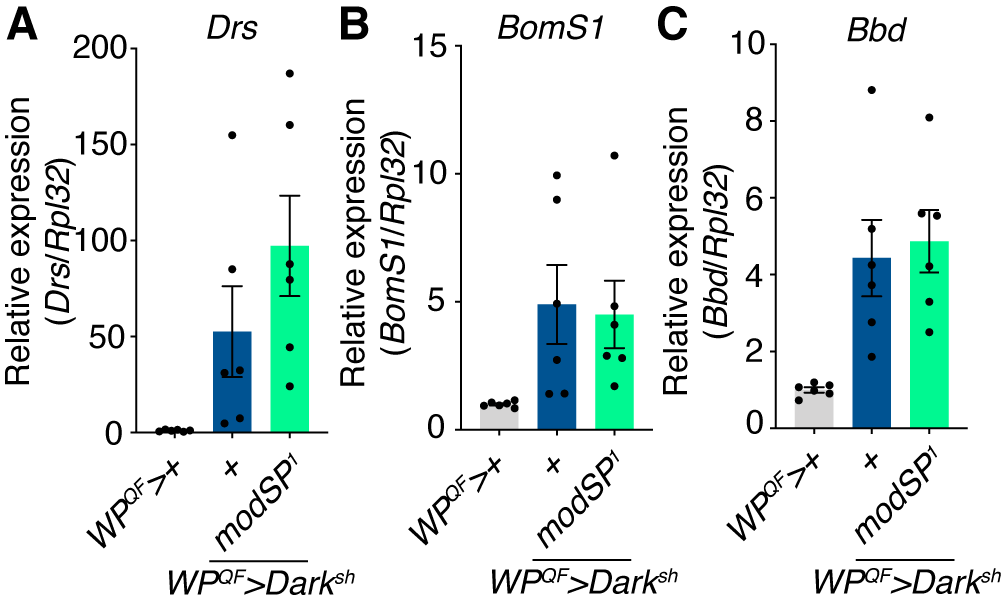

Supplement: S6 Fig — (A-C) Quantitative RT-PCR of Drs (A), BomS1 (B), and Bbd (C) in the abdominal cuticles of control (WPQF>+), apoptosis-deficient (WPQF>Darksh), or apoptosis-deficient with modSP1 background male flies raised on an antibiotics-supplemented diet. n = 6. Data are mean with SEM. Each dot represents a replicate. (TIF) [file pgen.1010761.s006.tif]

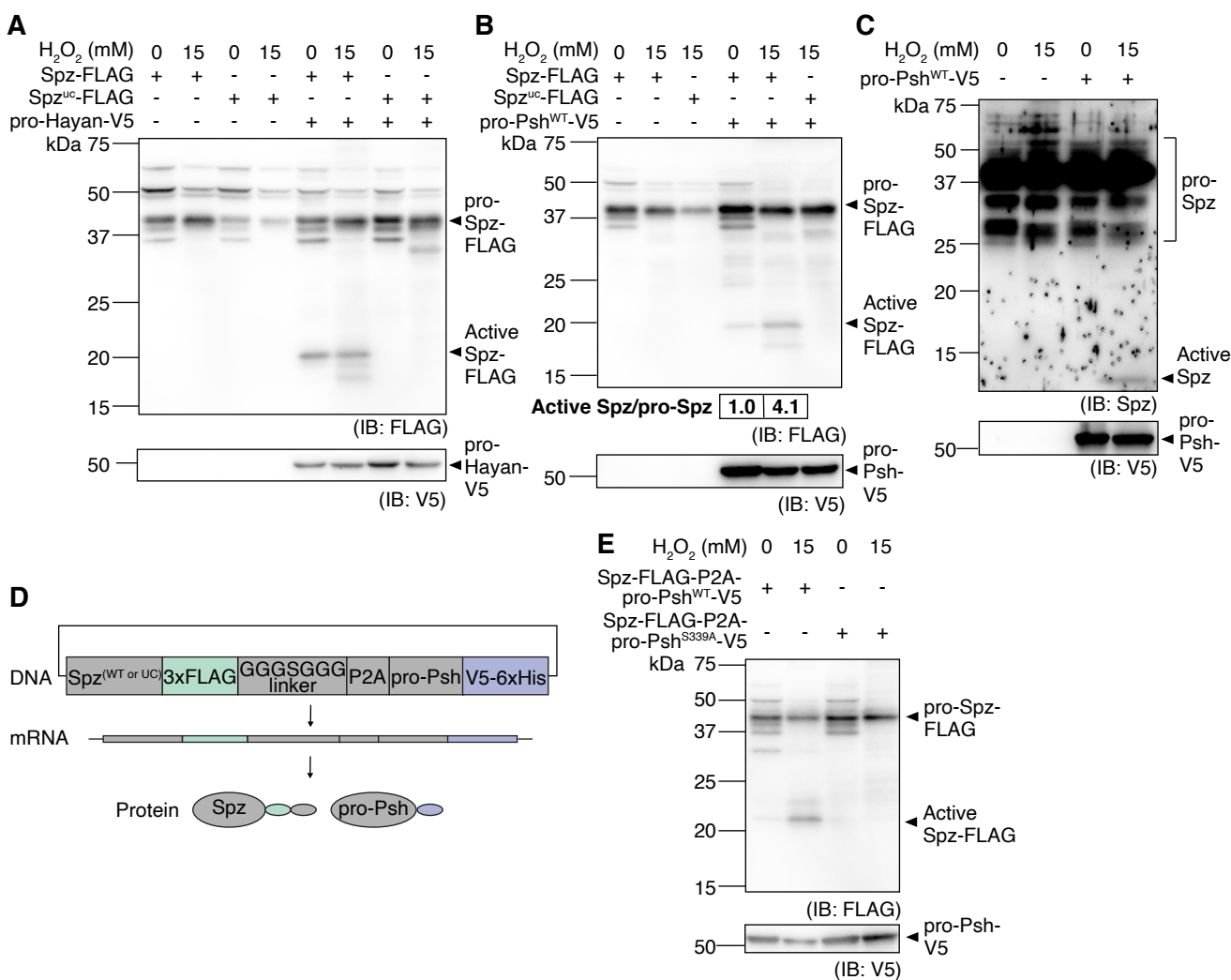

Supplement: S7 Fig — (A, B, and E) Western blotting of S2 cell lysates against FLAG-tagged Spz (anti-FLAG antibody) and V5-tagged pro-Hayan (A) or pro-Psh (B and E) (anti-V5 antibody). (A) S2 cells were transfected with either pMT-Spz-3xFLAG or pMT-Spzuc-3xFLAG and pMT-pro-Hayan-PA-V5. H2O2 treatment of 15 mM for 18 h. (B) S2 cells were transfected with either pMT-Spz-3xFLAG or pMT-Spzuc-3xFLAG and pMT-pro-PshWT-V5. H2O2 treatment of 15 mM for 18 h. The signal ratio of active Spz (~20 kDa)/pro-Spz (~37 kDa) was approximately 4-fold greater in the H2O2 treated condition, calculated using FUSION SOLO. 7S. EDGE software (Vilber-Lourmat). (C) Western blotting of S2 cell lysates against Spz (anti-Spz C106 antibody) and V5-tagged pro-Psh (anti-V5 antibody). S2 cells were transfected with pMT-pro-PshWT-V5. H2O2 treatment of 15 mM for 18 h. (D) Schematic representation of the plasmid tandemly bearing the coding sequences of Spz-3xFLAG and pro-Psh-V5-6xHis with the GGGSGGG linker and viral P2A sequence between them. After transcription, a single mRNA is translated with 2A-mediated hydrolysis, independently resulting in the production of both Spz and pro-Psh protein. (E) S2 cells were transfected with pMT-Spz-3xFLAG-P2A-pro-PshWT or S339A-V5. H2O2 treatment of 15 mM for 18 h. (PDF) [file pgen.1010761.s007.pdf]

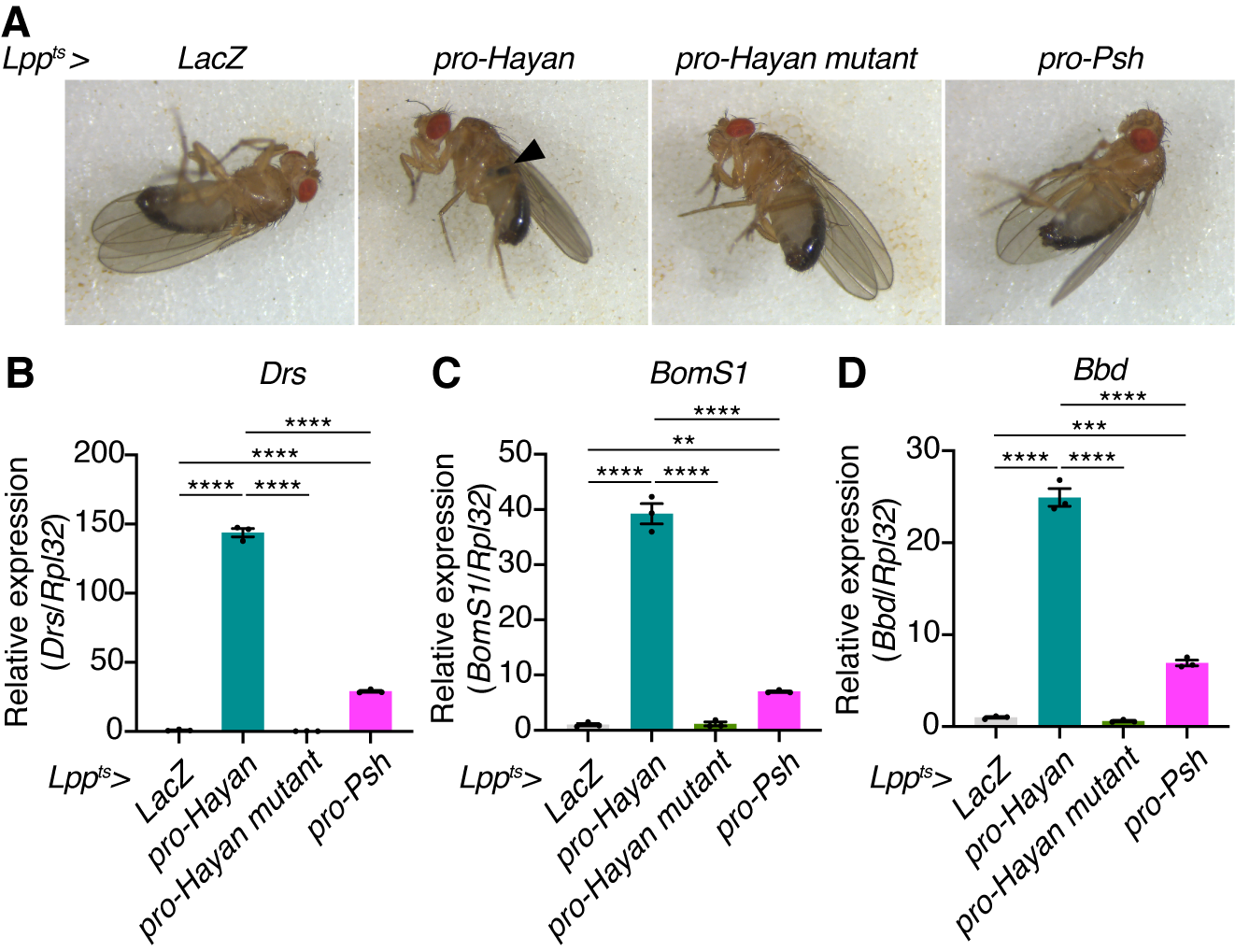

Supplement: S8 Fig — (A) Representative images of adult male flies overexpressing LacZ, pro-Hayan, pro-Hayan mutant, or pro-Psh by Lpp-Gal4, tub-Gal80ts (Lppts). Flies were reared at 18°C until 2–3 days after eclosion, then reared at 29°C for 48 h. An arrowhead indicates a melanotic mass. (B–D) Quantitative RT-PCR of Drs (B), BomS1 (C), and Bbd (D) in the whole body of male flies overexpressing LacZ, pro-Hayan, pro-Hayan mutant, or pro-Psh by Lpp-Gal4, tub-Gal80ts (Lppts). n = 3. Data are mean with SEM. Each dot represents a replicate. Statistical analysis was performed using one-way ANOVA with Tukey’s multiple comparison test. **: P < 0.01; ***: P < 0.001; ****: P < 0.0001. (TIF) [file pgen.1010761.s008.tif]

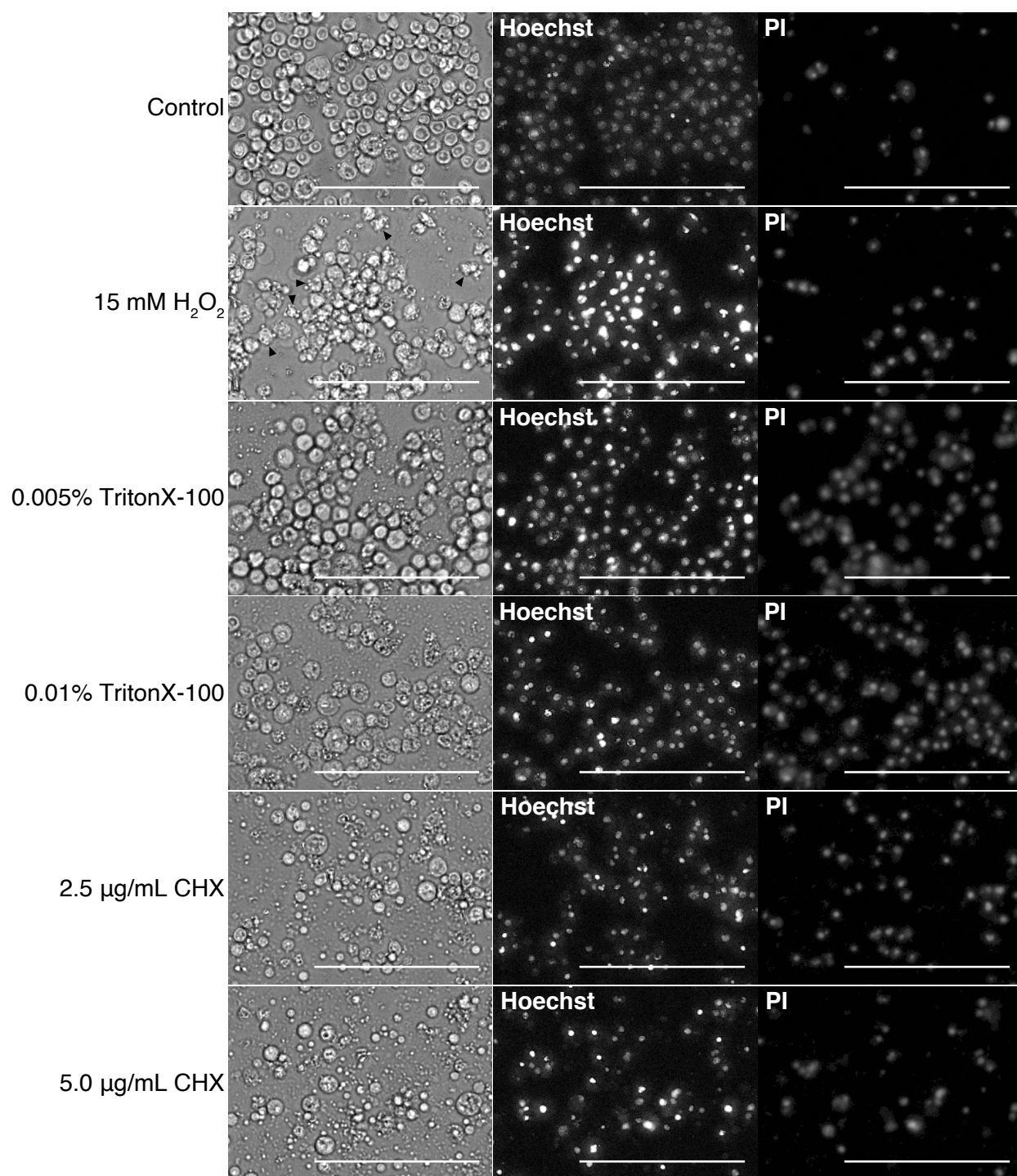

Supplement: S9 Fig — Representative images of S2 cells with Hoechst and PI staining. S2 cells were transfected with pMT-Spz-3xFLAG and pMT-pro-PshWT-V5. H2O2 treatment of 15 mM, TritonX-100 treatment of 0.005 or 0.01%, or CHX treatment of 2.5 or 5.0 μg/mL for 18 h. H2O2-treated S2 cells showed apoptotic traits, such as membrane blebbing (arrowheads) and nucleus condensation (Hoechst stain). TritonX-treated cells showed the increase in cell membrane permeability (PI). CHX-treated cells showed apoptotic traits, such as apoptotic body formation and nucleus condensation (Hoechst stain). Scale bars: 100 μm. (PDF) [file pgen.1010761.s009.pdf]

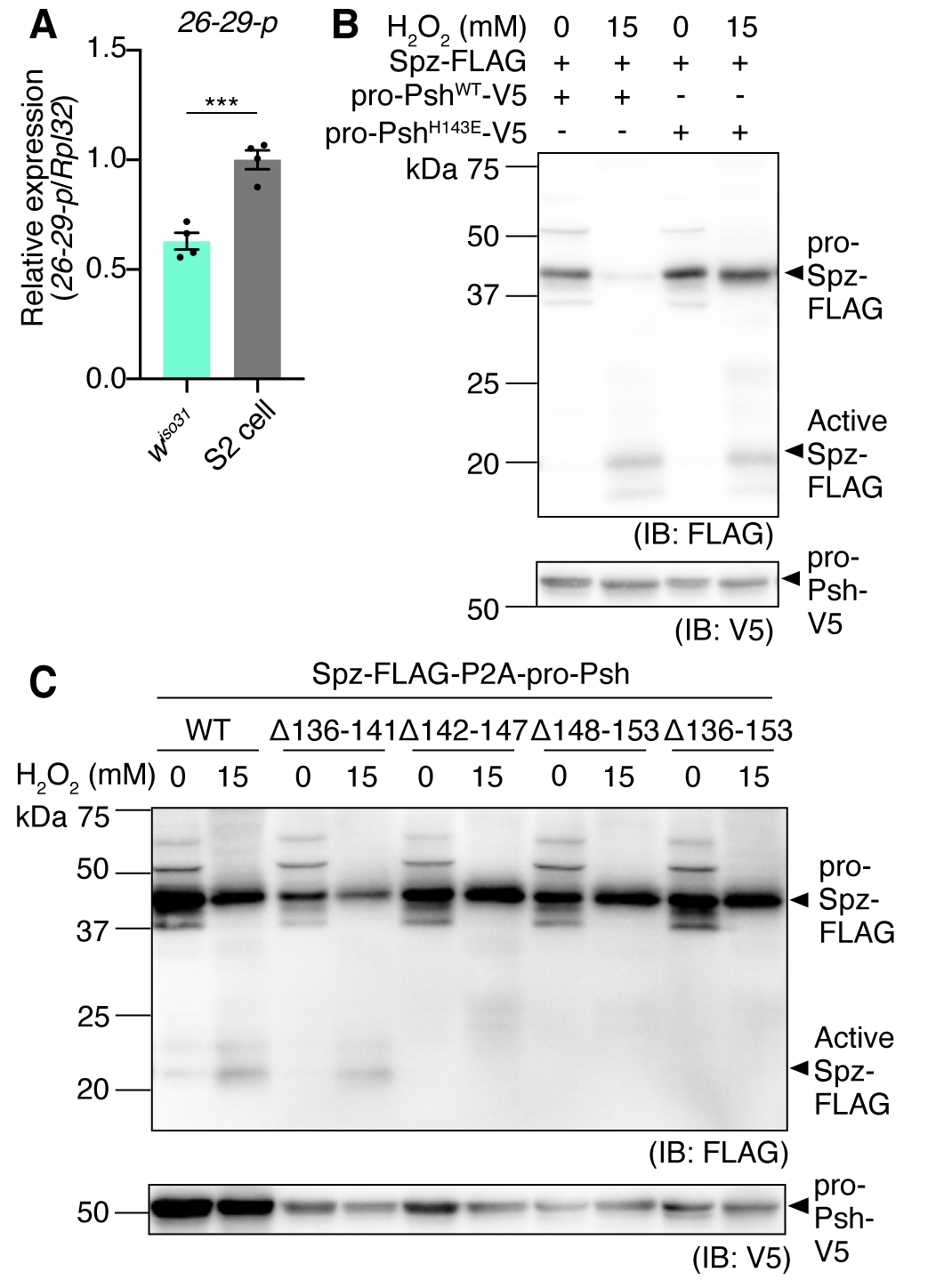

Supplement: S10 Fig — (A) Quantitative RT-PCR of 26-29-p in the whole body of control (wiso31) male fly and S2 cell samples. n = 4. Data are mean with SEM. Each dot represents a replicate. Statistical analysis was performed using two-tailed Welch’s t test. ***: P < 0.001. (B and C) Western blotting of S2 cell lysates against FLAG-tagged Spz (anti-FLAG antibody) and V5-tagged pro-Psh (anti-V5 antibody). (B) S2 cells were transfected with pMT-Spz-3xFLAG and pMT-pro-PshWT or H143E-V5. H2O2 treatment of 15 mM for 18 h. (C) S2 cells were transfected with pMT-Spz-3xFLAG-P2A-pro-PshWT, Δ136–141, Δ142–147, Δ148–153, or Δ136-153-V5. H2O2 treatment of 15 mM for 18 h. (TIF) [file pgen.1010761.s010.tif]

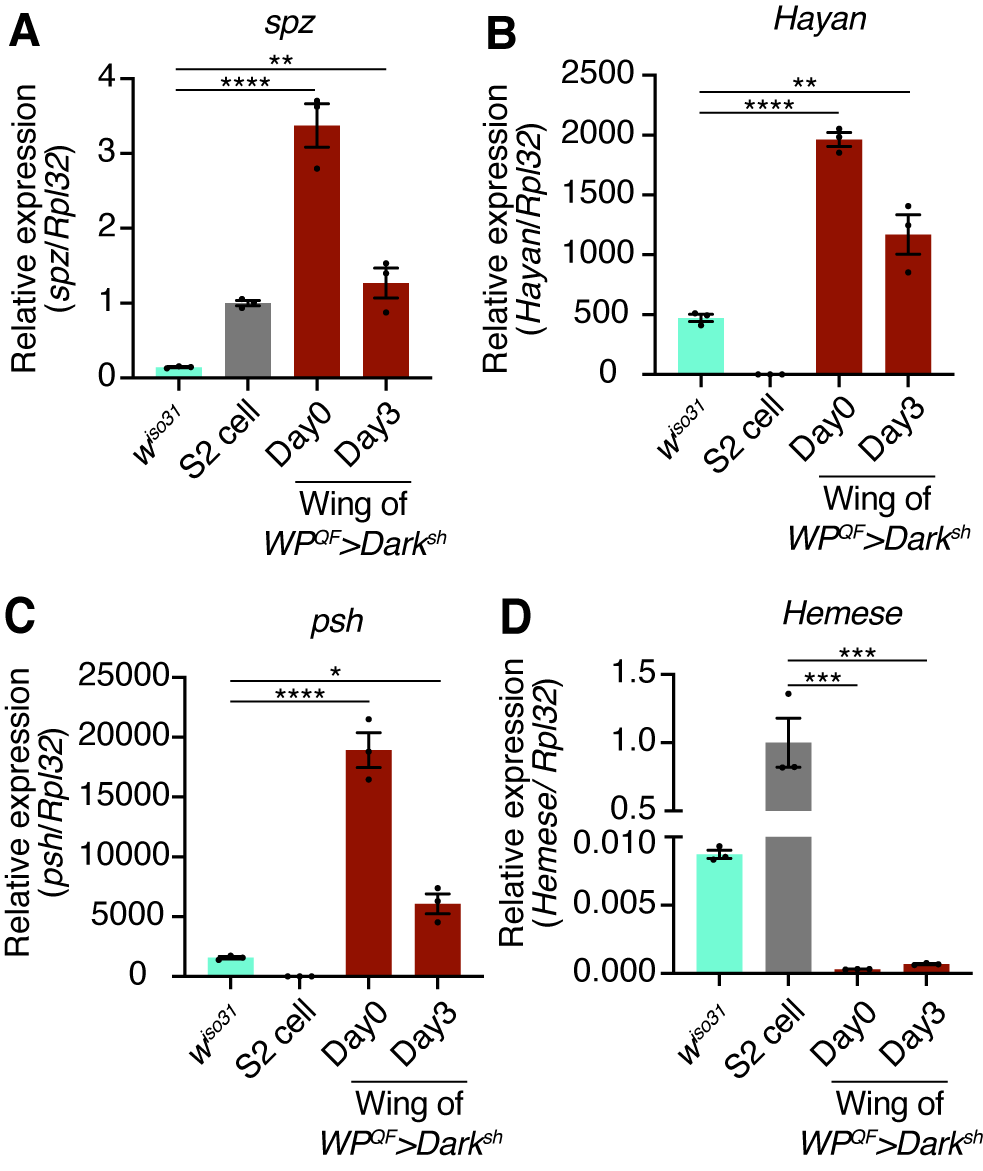

Supplement: S11 Fig — (A–D) Quantitative RT-PCR of spz (A), Hayan (B), psh (C), and Hemese (D) in the whole body of control (wiso31) male fly, S2 cell samples, and wings of apoptosis-deficient male flies 0 or 3 days after eclosion. n = 3. Data are mean with SEM. Each dot represents a replicate. Statistical analysis was performed using one-way ANOVA with Tukey’s multiple comparison test. *: P < 0.05; **: P < 0.01; ***: P < 0.001; ****: P < 0.0001. (TIF) [file pgen.1010761.s011.tif]

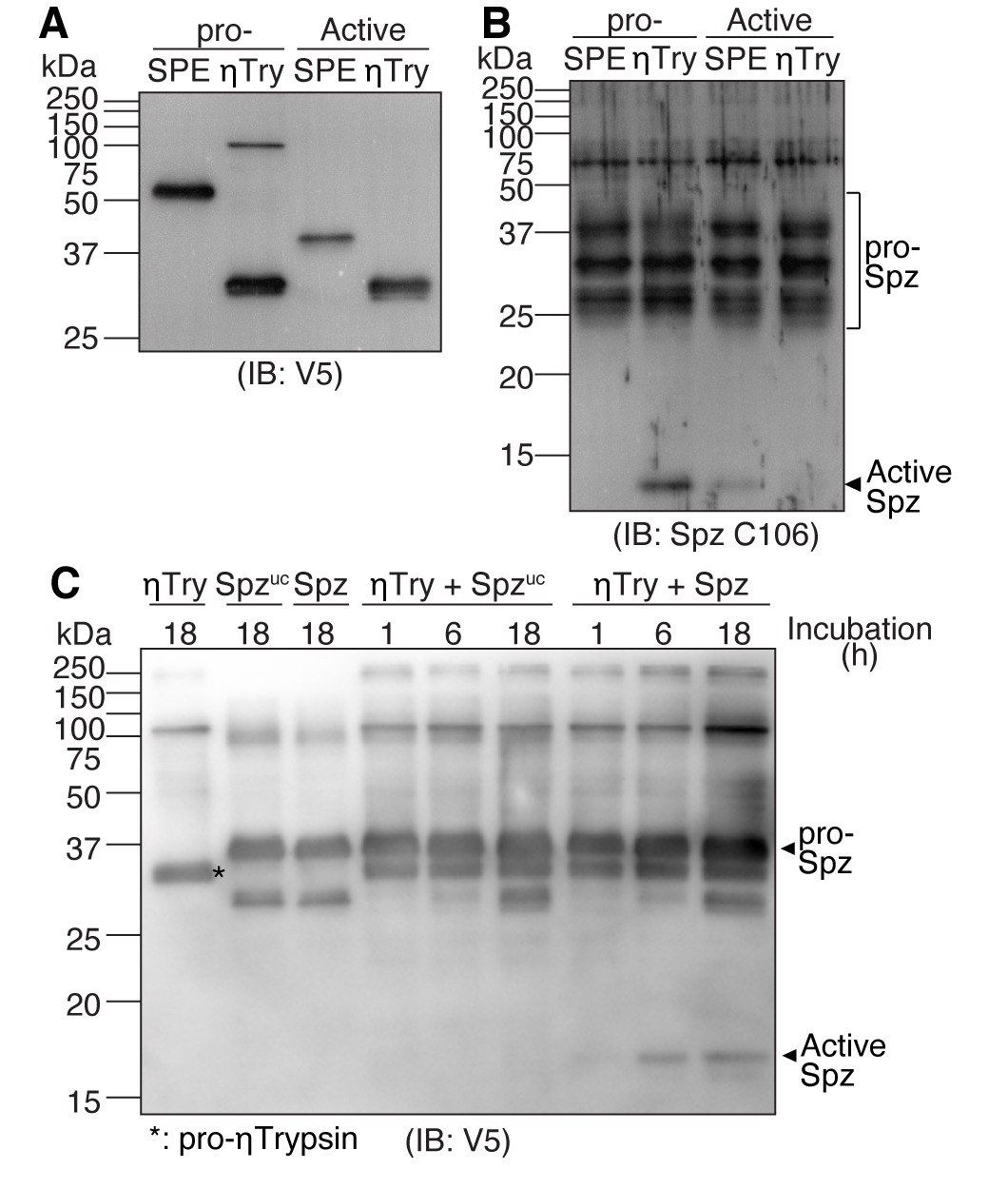

Supplement: S12 Fig — (A-C) Western blotting of S2 cell lysates against V5-tagged serine proteases (anti-V5 antibody) (A), Spz (anti-Spz C106 antibody) (B), and V5-tagged ηTrypsin and Spz (anti-V5 antibody) (C). (A and B) S2 cells overexpressed pro-SPE, pro-ηTrypsin, the catalytic domain of SPE, or the catalytic domain of ηTrypsin. Spz cleavage was observed when pro-ηTrypsin or the catalytic domain of SPE was overexpressed. (C) Lysates of S2 cells overexpressing pro-ηTrypsin, SpzWT, and Spzuc were collected, and Spz-containing lysates were mixed with pro-ηTrypsin-containing lysates. Incubation at 25°C for 1, 6, or 18 h. When SpzWT was incubated with pro-ηTrypsin, active Spz was observed 6 and 18 h after incubation, suggesting that Drosophila ηTrypsin can cleave Spz in a similar manner to mammalian trypsins. Notably, while pro-ηTrypsin is catalytically active, the putative active form ηTrypsin does not show catalytic activity against Spz, different from other standard serine proteases that need to be cleaved for their activation. This uniqueness of ηTrypsin is potentially due to the shortness of the pro-domain of ηTrypsin (the pro-domain of SPE is composed of 107 amino acids, while that of ηTrypsin is composed of 5); thus, the putative pro-domain of ηTrypsin is also required for the active conformation of ηTrypsin. (TIF) [file pgen.1010761.s012.tif]

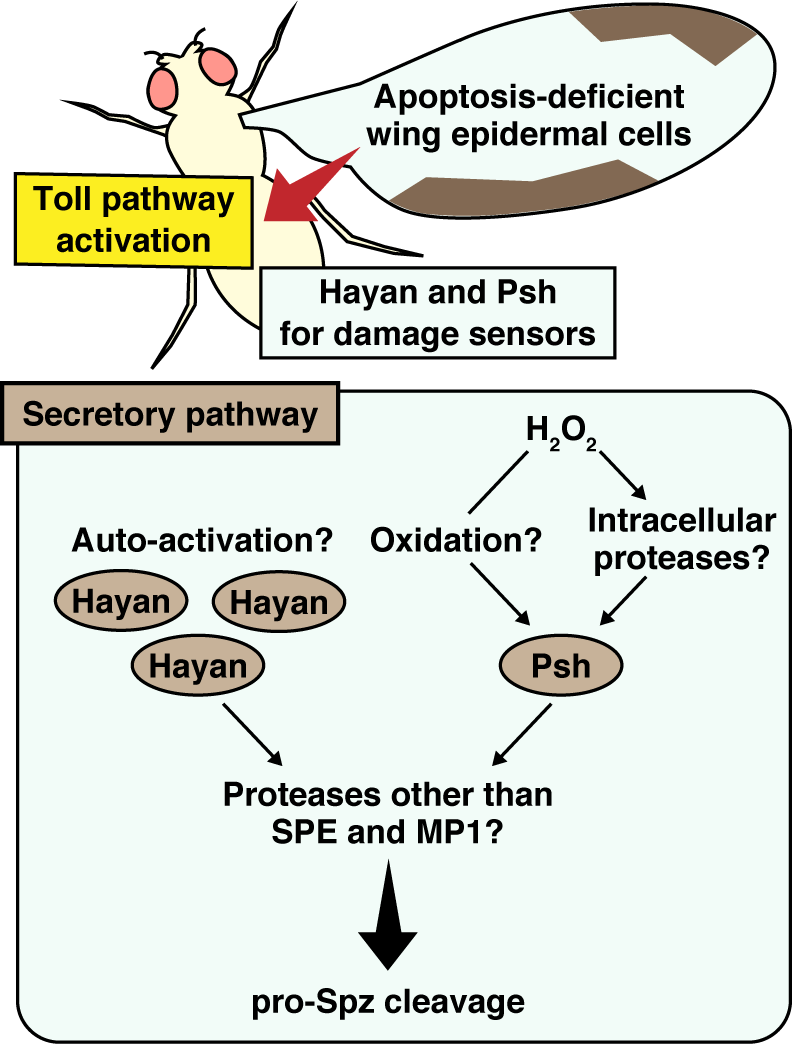

Supplement: S13 Fig — Hayan is auto-activated upon induction of its expression and Psh is activated by H2O2 in the secretory pathway of necrotic wing epithelia. Activated Hayan and Psh activate unknown SPs, and then pro-Spz is cleaved in the secretory pathway. (TIF) [file pgen.1010761.s013.tif]
