# Supplementary material for: Damage sensing mediated by serine proteases Hayan and Persephone for Toll pathway activation in apoptosis-deficient flies
Source: PLoS Genet. 2023 Jun 15;19(6):e1010761. doi: 10.1371/journal.pgen.1010761 (PMC10270351; doi:10.1371/journal.pgen.1010761)
Supplement: Attachment — Submitted filename: Response to reviewers_submit.pdf [file pgen.1010761.s021.pdf]

Cover Letter

Dear Editor,

We are pleased to submit our revised manuscript "Damage sensing mediated by serine proteases Hayan and Persephone for Toll pathway activation in apoptosis-deficient flies" to *PLOS Genetics*. We are grateful for the constructive comments from each of the reviewers. We have implemented their helpful suggestions and the manuscript has been revised according to their comments. The revised portions of the manuscript are written in red. Please see our point-by-point responses in the rebuttal letter.

Major changes:

We added new data suggested by the reviewers.

Expression levels of Toll target genes in apoptosis-deficient flies with *SPE, MP1* double mutant backgrounds were investigated (Fig 4A–C).

The involvement of 26-29-p, a Drosophila cathepsin, in Psh activation upon H_2O_2 treatment was investigated (Fig 6A–C and S10A and B Figs).

Expression levels of Toll target gens were compared between apoptosis-deficient flies and control flies with septic injury with *Micrococcus luteus* (S1A–C Figs).

Expression levels of serine protease genes were investigated in *Drosophila* S2 cells (S4A–E).

Expression levels of Toll target genes with septic injury with *M. luteus* were investigated in *SPE* single mutants, *MP1* single mutants, and *SPE, MP1* double mutant flies (S5A–C).

Expression levels of Toll target genes were investigated upon overexpression of pro-Hayan and pro-Psh *in vivo* (S8A–D).

In addition, we added new following data that support our conclusion.

The expression levels of Toll target genes were investigated when peroxide was scavenging by overexpression of Catalase (Fig 7B–E).

Spz cleavage upon overexpression of Psh bearing the deletions in the disordered domain was investigated (S10C and D Figs).

The expression levels of *spz*, *Hayan*, *psh*, and *Hemese* were investigated in wings of apoptosis-deficient flies (S11A–D Figs).

Now that we have addressed all the concerns from the reviewers, we believe our manuscript has improved enough to create a greater impact for the wide audience of this journal.

Thank you in advance for your kind consideration.

Sincerely yours,

Masayuki Miura, Ph. D.

Department of Genetics, Graduate School of Pharmaceutical Sciences,

The University of Tokyo, 7-3-1 Hongo, Bunkyo-ku, Tokyo 113-0033, Japan

Tel: +81-3-5841-4860, Fax: +81-3-5841-4867

E-mail: miura@mol.f.u-tokyo.ac.jp

Reviewer's Responses to Questions

Comments to the Authors:
Please note here if the review is uploaded as an attachment.

Reviewer #1: This is a very interesting study that analyzes the serine protease pathway leading to Toll activation in a Drosophila model of sterile stimulation. Although, many connections have been previously shown by other studies, this article links many disparate observations and clarify some important points combining both in vivo and cell culture approaches. Thus, this article is an important addition to the field of Drosophila immunity and wound healing.
I have however some recommendations and suggestions to improve the impact of this article.

Major recommendations
1) Figure 1A: it would have been interesting to know what is the level of Drs expression in the Dark background compared not only to unchallenged flies but also to flies infected with a Gram-positive bacterium (M. luteus). Just to have an idea of the range of activation of the Toll pathway. The authors could simply write the level of Drs expression in apoptosis deficient background represent x% of the level of flies infected with a G+. This will allow the community to estimate the level of Toll pathway activation in their system.

We would like to thank the Reviewer for their time and helpful comments in improving our manuscript. We really appreciate your first comment. We compared the expression levels of the Toll target genes *Drosomycin* (*Drs*), *Bomamin Short 1* (*BomS1*), and *Bombardier* (*Bbd*)) in apoptosis-deficient flies and flies infected with *Micrococcus luteus* (*M. luteus*) (S1A–C Figs). The level of expression of each gene in apoptosis-deficient flies was approximately 50–60% of that in flies infected with *M. luteus*. We have clarified this in the manuscript between **lines 184–187.**

“In apoptosis-deficient flies, expression levels of Toll target genes, including *Drosomycin* (*Drs*), *Bomanin Short 1* (*BomS1*), and *Bombardier* (*Bbd*), were approximately 50–60% of those in flies infected with *Micrococcus luteus*, a gram-positive bacterium (S1A–C Figs) (**lines 184–187**).”

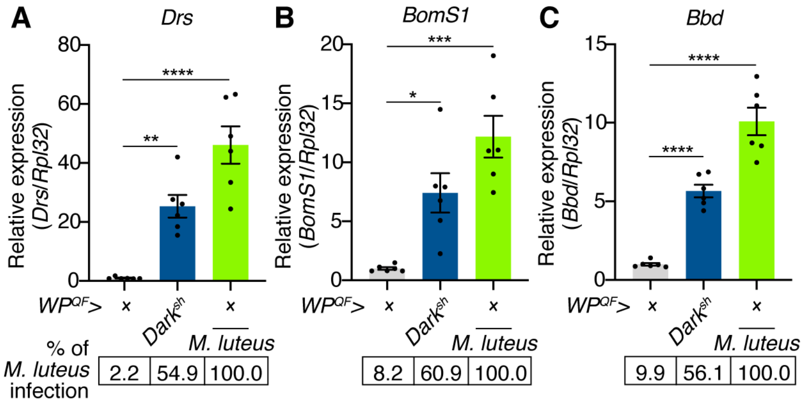

S1A–C Figs

2) The claims that SPE and MP1 redundantly contribute to Spz cleavage should be tested by using a SPE, MP1 double mutants. We should to know if spe, MP1 double mutants display a stronger impairment of Toll pathway activation than SPE or MP1 single mutants in their model of sterile inflammation and upon septic injury. If this is too challenging to combine, MP1, SPE with the genetic elements inducing apoptosis, the authors could inject ROS.

We took your suggestion very seriously and performed experiments with *SPE*, *MP1* double mutants in infectious or apoptosis-deficient condition (S5A–C Figs and Fig 4A–C). Please see our results and discussions in **lines 354–363 and 547–592**.

"Unexpectedly, *SPE*^{SK6} or *MP1*^{SK6} single mutants, as well as *SPE*^{SK6} + *MP1*^{SK6} double mutants, showed Toll pathway activation in apoptosis-deficient flies that was comparable to (in *SPE*^{SK6} and *SPE*^{SK6} + *MP1*^{SK6}) or even greater than (in *MP1*^{SK6}) that of the controls (Figs 4A–C). In contrast, upon septic injury with *M. luteus*, the expression of Toll target genes was suppressed in *SPE*^{SK6} but not in *MP1*^{SK6} single mutants, and *SPE*^{SK6} + *MP1*^{SK6} double mutants showed stronger suppression of Toll target genes than *SPE*^{SK6} single mutants (S5A–C Figs). These results suggest that, although *SPE* and *MP1* redundantly contribute to Toll activation upon infection through Spz cleavage, they are not the SPs significantly involved in Spz cleavage in apoptosis-deficient flies. (**lines 354–363**)"

"Indeed, we found that *SPE*, *MP1* double mutants show a stronger suppression of Toll

target genes upon infection than *SPE* single mutants, providing further evidence of the redundant function of MP1 for Spz cleavage upon infection. In contrast, *SPE*, *MP*1 double mutants showed Toll activation similar to controls in apoptosis-deficient flies, implying that *SPE* and *MP*1 are not the significant SPs involved in Spz cleavage in this case. These results raise the question of how Spz cleavage occurs in apoptosis-deficient flies if SPE and MP1 do not play an important role. One hypothesis is that another hemolymph protease, other than SPE and MP1, cleaves Spz downstream of Hayan and Psh. Potentially, the proteomic analysis could not identify proteases present in small amounts.

Based on our results, Toll activation in apoptosis-deficient flies relies upon Hayan and Psh. If activation of Hayan and Psh occurs in the hemolymph of apoptosis-deficient flies, subsequent Spz cleavage by SPE and MP1 should occur in the hemolymph as seen in infection. However, the *SPE*, *MP1* double mutations did not impair Toll activation in apoptosis-deficient flies. Thus, another proposed mechanism could be that Spz cleavage that depends on Hayan and Psh occurs intracellularly in response to necrotic damage. This hypothesis could be indirectly supported by the following evidence. First, in the present study, pro-Hayan overexpression or pro-Psh overexpression with H_2O_2 treatment induces intracellular Spz cleavage in S2 cells. Moreover, upregulation of *Hayan* gene expression occurs in hemocytes upon epidermal injury [6]. In addition, a previous study reported that H_2O_2 produced at wound sites is efficiently diffused into hemocytes through Prip, a channel protein [6]. Thus, these support the idea that, in Spz-producing cells, pro-Hayan or pro-Psh can be intracellularly activated in response to tissue damage. Although MP1 is required for Spz cleavage in S2 cells, it is possible that *in vivo* another SP becomes activated downstream of Hayan or Psh to cleave Spz in hemocytes recruited to damaged sites or in neighboring cells of damaged tissues. Indeed, we found that ηTrypsin, a *Drosophila* trypsin, could induce Spz cleavage in S2 cells (S12A–C Figs), suggesting that certain trypsin-like SPs can cleave Spz intracellularly.

Furthermore, it has been reported that Spz belongs to the neurotrophin protein family, sharing the conserved cystine-knot structure with mammalian neurotrophins, such as brain-derived neurotrophic factor (BDNF) or nerve growth factor (NGF) [47,58,59]. In mammals, BDNF is synthesized as the precursor pro-BDNF and matures by protease-mediated cleavage, a process reminiscent of pro-Spz activation [60]. Importantly, it has been reported that processing of pro-BDNF not only occurs extracellularly via proteases such as plasmin, but also occurs intracellularly in secretory pathways (trans-Golgi network and secretory vesicles) via furin and proprotein

convertases [61–63]. Given the similarity between Spz and BDNF, Spz may also be cleaved intracellularly to achieve maturation in response to damage. While this hypothesis is still at a conceptual stage, future investigation into whether Spz cleavage also occurs intracellularly upon tissue damage *in vivo*, along with which proteases are involved, is worthwhile. (**lines 547–592**)"

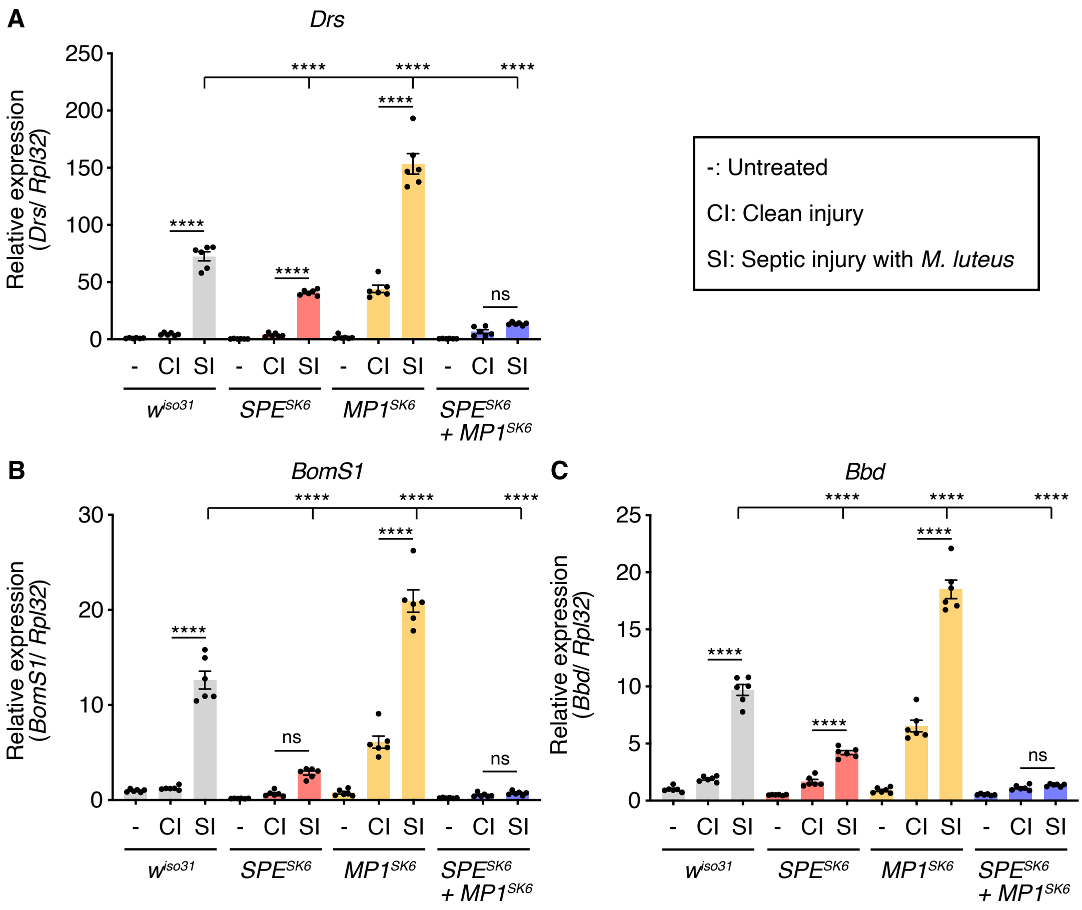

S5A–C Figs

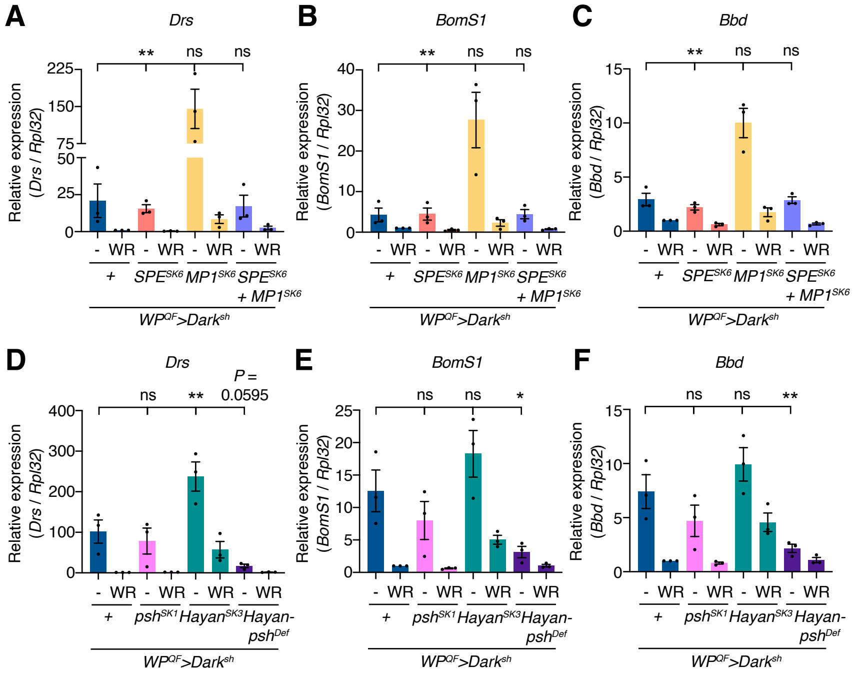

Figs 4A–F

Suggestions to improve the paper

I have two suggestions that could greatly improve the impact of the manuscript, but could also be considered as too much of extra-work.

1) Concerning Figure 3: The authors reports that expression of an activated forms of hayan or PSH can activate the Toll pathway through MP1. It would be great to know if this is the case in vivo in flies as the current knowledge suggests that only SPE is required. In short, do flies over-expressing activated forms of Hayan or PSH activate the Toll pathway? And if yes, it is really through MP1?

We greatly appreciate your first suggestion. Due to time constraints, it was difficult to perform experiments using active forms of Hayan or Psh; however, we did examine whether overexpression of pro-Hayan or pro-Psh activates the Toll pathway *in vivo* (S8A–D Figs). Please see our results and discussions in **lines 402–411**.

"We also investigated whether overexpression of pro-Hayan or pro-Psh activates the Toll pathway *in vivo*. Each gene was overexpressed in adult fat bodies by using *Lpp-Gal4* in combination with *tub-Gal80^{ts}*, where a temperature-sensitive Gal80

(Gal80^{ts}) inhibits Gal4 at lower temperature (18 °C). Overexpression of pro-Hayan, but not catalytic-dead pro-Hayan (pro-Hayan mutant; [50]) or pro-Psh, induced melanization in adult flies, as reported in a previous study using *Drosophila* larvae [50] (S8A Fig). Overexpression of pro-Hayan or pro-Psh, but not pro-Hayan mutant, activated the Toll pathway, while pro-Hayan induced stronger activation than pro-Psh (S8B–D Figs). This result is consistent with the results of experiments using S2 cells and support the idea that Hayan is more easily auto-activated than Psh when overexpressed. (**lines 402–411**)"

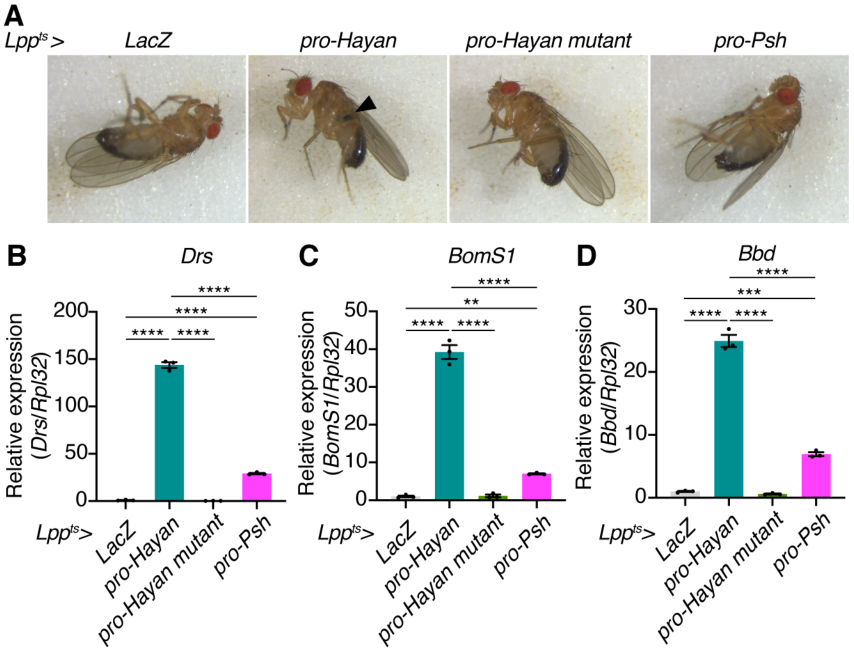

S8A–D Figs

2) It would have been great to analyze the activation of the Toll pathway in both their model of sterile injury and upon septic injury by a gram-positive bacterium in Hayan, MP1 and Psh, MP1 double mutants flies to better characterize the relation between these serine protases.

Once again, we appreciate your suggestion. Unfortunately, due to time constraints, we did not perform the experiment you suggested. From our present results, MP1 is required for Spz cleavage downstream of Hayan and Psh in S2 cells and is likely to have a redundant function to that of SPE. This was

suggested since *SPE*, *MP1* double mutants showed more impairment of Toll activation upon infection with *M. luteus* than *SPE* single mutants (S5A–C Figs). These results further suggest that MP1 contributes to Spz cleavage upon infection with Gram-positive bacteria downstream of Hayan and Psh. Thus, we think that *Hayan, MP1* and *Psh, MP1* double mutants will not show the impairment of the Toll pathway upon infection because SPE can still function to cleave Spz. Moreover, *Hayan, MP1* and *Psh, MP1* double mutants will also not show impairment of the Toll pathway upon necrotic tissue damage because the *SPE*, *MP1* double mutants did not show impairment of Toll activation in apoptosis-deficient flies.

Minor recommendation

Line 81: add the fat body in addition to hemocytes and LG.

We appreciate and agree with your comment and have added the fat body as an immune responsive organ on **lines 84–86**.

"Upon clean injury to the larval or adult epidermis, Toll signaling is activated in fat bodies, circulating hemocytes, and the larval lymph gland (hematopoietic organ) **(lines 84–86)**"

Lines 88-93: maybe worth to mention the work on spz5

We appreciate your comment. As you mentioned, it has been previously reported that Spz5 behave as a ligand for Toll-1 *in vivo* and *in vitro* (Nonaka et al., Biochem Biophys Res Commun., 2018; Chowdhury et al., J Biol Chem., 2019).Therefore, we have now mentioned Spz5 as a Toll-1 ligand on **lines 98–99** .

"Toll receptors are activated by interacting with the protein ligand Spätzle (Spz) or Spz5 [17–19]. (**lines 98–99**)"

Line 157: The authors should provide a bit more information on their apoptosis deficient system.

We appreciate and agree with your comment. We have added a brief explanation of the mechanism of caspase activation in apoptosis and the use of the Dark knockdown using QF/QUAS system in our study. Please see **lines 168–178**.

"During *Drosophila* development, WECs are cleared from the adult wings by undergoing apoptosis within 3 h after eclosion [36,37]. This apoptosis is mediated by a cysteine protease, caspase, that becomes activated upon making a protein complex, apoptosome [38]. In *Drosophila melanogaster*, Dronc (Mammalian orthologue is Caspase-9) and Death-associated APAF1-related killer (Dark) (Mammalian orthologue is Apaf-1) form the apoptosome [39]. Here, the caspase activation in WECs was blocked by knockdown of *Dark* using the QF/QUAS system, as reported in our previous studies [11,12]. Briefly, the *wing pouch (WP) -QF2* driver overexpresses a short hairpin RNA targeting *Dark* specifically in WECs by combination with *QUAS-Dark-sh*. This knockdown of *Dark* efficiently blocks apoptosis of WECs, leading to necrotic cell death characterized by cellular membrane permeabilization [11] (**lines 168–178**)."

Line 193: So far, Bomanins are not AMPs but rather host defense peptides (no detected microbicidal activity)

We would like to apologize for any confusion. We have revised our understanding of Bomanins and have rephrased the sentence (**lines 236–237**). As you mentioned, some synthesized Bomanins do not show a fungicidal effect *in vitro* (Lindsay et al., J Innate Immun., 2018), and a previous report has also described the role of BomS6 in host resilience to infection through protection against a microbial toxin (Xu et al., EMBO Rep., 2022).

"Bomanins, a type of host defense peptides [44] (**lines 236–237**)"

Line 203-204: the statement is too strong. Previous studies have shown that cleaved form of spz activates the Toll pathway (ex. Delotto and Delotto 1998). Just rephrase.

We appreciate and agree with your assessment. We have omitted the strong phrasing as suggested (**line 247–249**).

"Two SPs, Easter and SPE, cleave Spz during embryogenesis and infection, respectively (17,41)~~; however, it has not been investigated whether Spz cleavage is essential for Toll activation in vivo~~. To test the requirement of Spz cleavage in apoptosis-deficient flies, (**lines 247–249**)"

Line 241: Are the SP secreted in the extracellular space or do they activate spz in the secretory pathway (Golgi?) in cells? I would have expected that SP cleaves Spz extracellularly.

We have addressed this important point further in the text (**lines 304–306**). We deduced that these overexpressed SPs were secreted via the secretory pathway, because we could detect the SPs in the culture medium by Western blotting (data not shown). Therefore, as you expected, we predicted that Spz cleavage can occur extracellularly. However, in our analysis (Fig. 3C), we collected cell lysate samples without medium and believe that Spz cleavage also occurs in intracellularly. Indeed, Yamamoto-Hino and Goto also detected Spz cleavage in S2 cell lysate overexpressing Spz-FLAG with active SPE or active MP1 (Yamamoto-Hino and Goto, Cell Struct Funct., 2016).

"Since S2 cells characteristically express Spz protein, the overexpressed active form of each SP should co-localize with the endogenous Spz and subsequently be able to cleave it in the secretory pathway [35]. (**lines 304–306**)"

Line 288: for clarification, mention that those pro-forms are not active

We appreciate your comments and have now clarified that pro-forms are putatively inactive (**lines 391–393**).

"Putatively inactive pro-Psh or pro-Hayan (isoform A), along with Spz, were overexpressed in S2 cells, and Spz cleavage in cell lysates was examined when S2 cells were treated with H_2O_2 (**lines 391–393**)"

Line 290: can the authors speculates why proHayan can cleave spz in S3B

Thank you for your suggestion. Please see our discussion in **lines 393–395 and 608–616**.

"Overexpression of pro-Hayan led to Spz cleavage with or without H_2O_2 treatment (S7A Fig), suggesting that pro-Hayan is auto-activated by overexpression, similar to modSP [28]. (**lines 393–395**)"

"Overexpression of pro-Hayan (isoform A) in S2 cells resulted in Spz cleavage, suggesting that pro-Hayan can be auto-activated, an activation mode reminiscent of that of the initiator protease modSP [28]. In our proteomic analysis, Hayan protein was undetectably low in control flies, yet was increased in apoptosis-deficient flies. This increasing trend was also observed in a previous study that reported upregulation of the *Hayan* gene in hemocytes upon clean injury to adult *Drosophila* [6]. These observations likely support the idea that more Hayan is produced upon tissue damage, and this increased Hayan is auto-activated like an initiator protease to trigger Toll activation and melanization in damaged sites. (**lines 608–616**)"

Line 305 and earlier; it would have been great to know which SP genes are endogenously expressed in S2 cells.

We appreciate and agree with your suggestion and have added to the text (**lines 315–318**). To examine the expression levels of SP genes in S2 cells, we performed quantitative RT-PCR of *SPE*, *MP1*, *easter*, *Hayan*, and *psh* in S2 cell and whole-body fly samples (S4A–E Figs). While the expression of *SPE*, *easter*, *Hayan*, and *psh* genes were markedly lower in S2 cells compared to those in whole-body fly samples, that of *MP1* gene was comparable.

"While the expression levels of *SPE*, *easter*, *Hayan*, and *psh* genes were markedly lower in S2 cells compared to those in whole-body fly samples, that of the *MP1* gene was comparable (S4A–E Figs). Consistent with the transcript level, SPE protein was not detected in the S2 cell lysate (Fig 3D) (**lines 315–318**)"

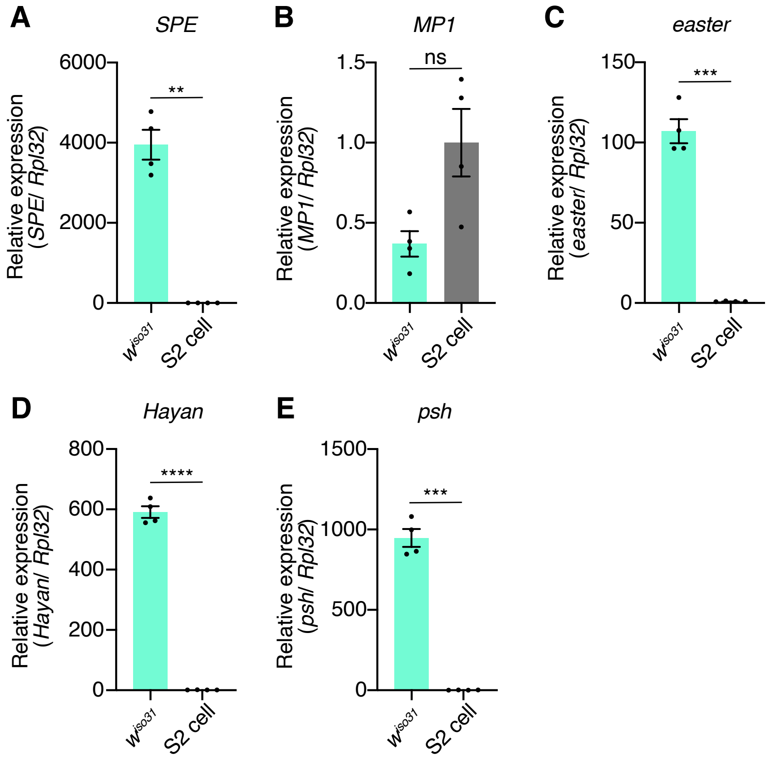

S4A–E Figs

Lines 369-370: it seems that the partially overlapping/redundant roles of Hayan and Psh has clearly been established by Dudzic 2019: Toll was not activated by proteases and septic injury in the double mutants flies.

We apologize for any misleading phrasing. Here, we would like to suggest that the redundant function of both Hayan and Psh should also be examined in the case of Toll activation upon larval epidermal injury, since the previous study (Evans et al., PNAS., 2022) only examined the involvement of Psh.

"However, there seems to be a discrepancy in the requirement of Psh for sterile activation of the Toll pathway because Toll activation upon clean injury to the larval epidermis is not suppressed in *psh* single mutants [7]. Hayan may redundantly contribute to this Toll activation upon larval injury because Hayan is involved in melanization upon injury [30,50]. Since the redundant functions of Hayan and Psh in Toll activation has been already established in a previous study [30] and confirmed in

the present study, investigation of whether Toll pathway activation upon larval injury is also suppressed in *Hayan* and *psh* double mutants is necessary. (**lines 598–605**)"

Lines 374: Does over-expression of Hayan sufficient to activate the Toll pathway. Is it consistent with results published by Nam et al., EMBO ?. Here again, it would have great to know if over-expression of Hayan is sufficient to activate the Toll pathway in vivo in flies.

We appreciate your comment. As we mentioned in "your suggestion 1)", we examined whether overexpression of pro-Hayan activates the Toll pathway in adult flies (S8A–D Figs). Please see our results and discussions in **lines 402–411**.

"We also investigated whether overexpression of pro-Hayan or pro-Psh activates the Toll pathway *in vivo*. Each gene was overexpressed in adult fat bodies by using *Lpp-Gal4* in combination with *tub-Gal80^{ts}*, where a temperature-sensitive Gal80 (Gal80^{ts}) inhibits Gal4 at lower temperature (18 °C). Overexpression of pro-Hayan, but not catalytic-dead pro-Hayan (pro-Hayan mutant; [50]) or pro-Psh, induced melanization in adult flies, as reported in a previous study using *Drosophila* larvae [50] (S8A Fig). Overexpression of pro-Hayan or pro-Psh, but not pro-Hayan mutant, activated the Toll pathway, while pro-Hayan induced stronger activation than pro-Psh (S8B–D Figs). This result is consistent with the results of experiments using S2 cells and support the idea that Hayan is more easily auto-activated than Psh when overexpressed. (**lines 402–411**)"

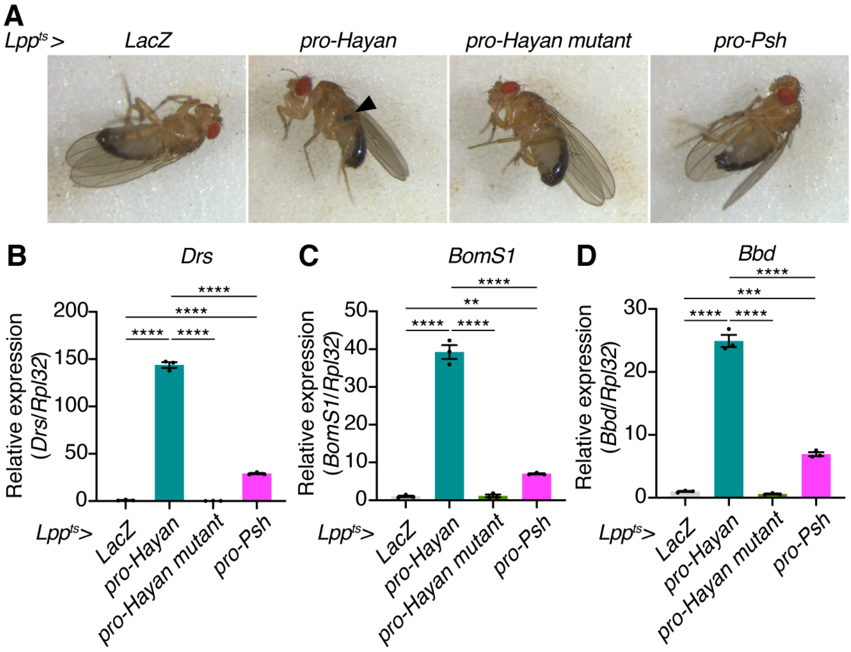

S8A–D

Line 292-396: The implication of lysosomes was not clear to me. Does the author mean that the activation of Psh by H2O2 take place inside a cell or is it linked to the release of proteases due to the breakage of lysosomes in the hemolymph that activated PSH extracellularly. Provide a bit more information.

We appreciate and agree with your comment. Given that cathepsin 26-29-p does not significantly contribute to Psh activation and that Psh is translocated in the endoplasmic reticulum, we now think that Psh activation by H2O2 likely occurs in the secretory pathway. We revised the sentence regarding the mechanism of Psh activation (**lines 627–633**).

"Since the unfolded disordered domain is susceptible to proteolysis [56,57], Psh is likely activated through cleavage within this domain by unknown proteases other than 26-29-p downstream of H_2O_2. Since Psh is translocated into the endoplasmic reticulum, it is possible that Psh activation in S2 cells occurs in the secretory pathway. Therefore, it is necessary to investigate the involvement of proteases in Psh processing through the secretory pathway. (**lines 627–633**)"

Line 424; I would remove the term pathogen.

We agree with your suggestion and have removed the term "pathogen" (**lines 664–666**).

"In conclusion, apoptosis defects in WECs lead to sterile activation of the Toll pathway through Hayan and Psh, establishing them as sensors for necrotic damage (S12 Fig). (**lines 664–666**)"

"In conclusion, apoptosis defects in wing epidermal cells lead to sterile activation of the Toll pathway~~, depending on the pathogen sensors~~ through Hayan and Psh, establishing them as ~~common~~ sensors for both infection and damage to tissues (S12 Fig). (**lines**)"

Reviewer #2: In this manuscript, Miura and coll. investigate activation of the Toll signaling pathway in drosophila in response to tissue damage. The manuscript is well written and I did not detect major problems in the experiments performed, or any major flaw in the interpretation of the results. Overall, the authors use a combination of genetics and biochemical experiments in S2 tissue culture cells to provide a refined model of the role of serine proteases acting upstream of the cytokine Spatzle (Spz) to activate Toll. This model emphasizes the role of Hayan and Persephone in a damage sensing pathway, in the absence of infection, and a more complex role than previously known for Melanization Protease 1 (MP1), which is able to cleave Spz in the S2 cell assay. While I enjoyed reading the manuscript, I could not help from feeling frustrated that the mechanism coupling ROS production to Psh activation is not addressed and one is left wondering about the possible involvement of the cathepsin 26-29p. Overall, I believe that this manuscript is a suitable candidate for publication in PLoS genetics provided the following points are addressed by the authors:

Major points

1) The role of the cathepsin 26-29p, which I believe is expressed in S2 cells, should be addressed.

First, we would like to thank the reviewer for their time and comments. We took your first suggestion very seriously and tested the role of cathepsin 26-29-p in

Spz cleavage (Figs 6A–C and S10A–D Figs). Please see **lines 457–469, 482–496, and 623–633** for our added results and discussion.

"Upon infection, pro-Psh is activated in two steps [32]. First, pro-Psh is cleaved at the bait region by a wide range of microbial proteases, and then it is further processed between His^{143} and Ile^{144} by a circulating endogenous cysteine cathepsin, 26-29-p, to become activated. Gene expression levels of 26-29-p in S2 cells were comparable to that in whole-body fly samples (S10A Fig). To determine whether 26-29-p also contributes to Psh activation upon H_2O_2 treatment, 26-29-p was knocked down by dsRNA treatment of S2 cells. However, Spz cleavage was still observed (Figs 6A and B). In a previous study, it was reported that a mutated Psh bearing a substitution of His^{143} with Glu^{143} (Psh^{H143E}) is resistant to cleavage by 26-29-p [32]. When pro-Psh^{H143E} was overexpressed, H_2O_2 still induced Spz cleavage in S2 cells (S10B Fig). In addition, Spz cleavage was also observed when pro-$Psh^{H143G,\ or\ H143A}$ was overexpressed (Fig 6C). These results suggest that the canonical cleavage of Psh by 26-29-p did not significantly contributes to Psh activation upon H_2O_2 treatment of S2 cells. (**lines 457–469**)"

"Based on the structure predicted by AlphaFold [54,55], the canonical cleavage site of Psh (His^{143} and Ile^{144}) is located in a disordered region (18 amino acids from Ser^{136} to Gly^{153}) between a helix and peptidase S1 domain (S10C Fig, adapted from AlphaFold DB: Q9VWU1). In general, disordered regions are susceptible to proteolysis [56,57]. To test the requirement of the disordered region for Psh-dependent Spz cleavage, pro-Psh carrying either a partial or complete deletion of the disordered region was overexperssed. For the partial deletions, 18 consecutive amino acids residues of the disordered region (Ser^{136} to Gly^{153}) were divided into three equal parts, and pro-Psh mutants carrying deletion of each 6 consecutive amino acids (Δ136-141, Δ142-147, or Δ148-153) were generated. When pro-$Psh^{\Delta136\text{-}141}$ was overexpressed, Spz cleavage was still observed, while overexpression of pro-Psh mutants with the other partial deletions (pro-$Psh^{\Delta142\text{-}147}$ or pro-$Psh^{\Delta148\text{-}153}$) or with the complete deletion (pro-$Psh^{\Delta136\text{-}153}$) did not show Spz cleavage (S10D Fig). These results suggest that upon H_2O_2 treatment, the disordered region from Ile^{142} to Gly^{153} plays an important role in Psh activation, potentially by undergoing cleavage by intracellular proteases. (**lines 482–496**)"

"A candidate cysteine cathepsin 26-29-p, which can process and activate Psh upon infection [32], was not significantly involved in Psh activation induced by H_2O_2 treatment of S2 cells. We also found that Psh mutants carrying deletion of the disordered domain between the CLIP- and peptidase S1 domains did not show Spz

cleavage upon H_2O_2 treatment. Since the unfolded disordered domain is susceptible to proteolysis [56,57], Psh is likely activated through cleavage within this domain by unknown proteases other than 26-29-p downstream of H_2O_2. Since Psh is translocated into the endoplasmic reticulum, it is possible that Psh activation in S2 cells occurs in the secretory pathway. Therefore, it is necessary to investigate the involvement of proteases in Psh processing through the secretory pathway. (**lines 623–633**)"

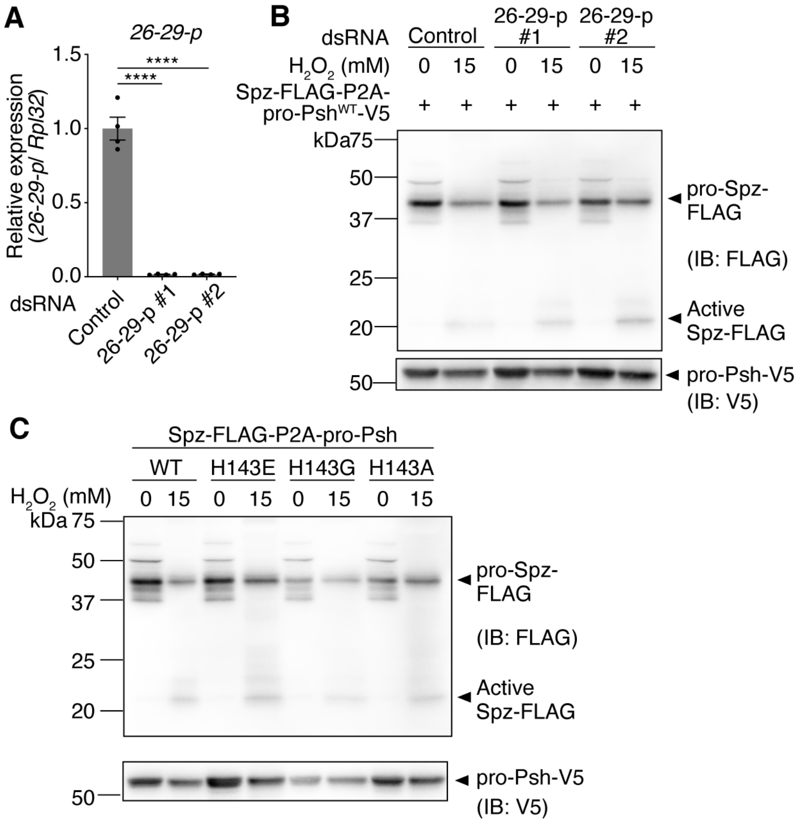

Figs 6A–C

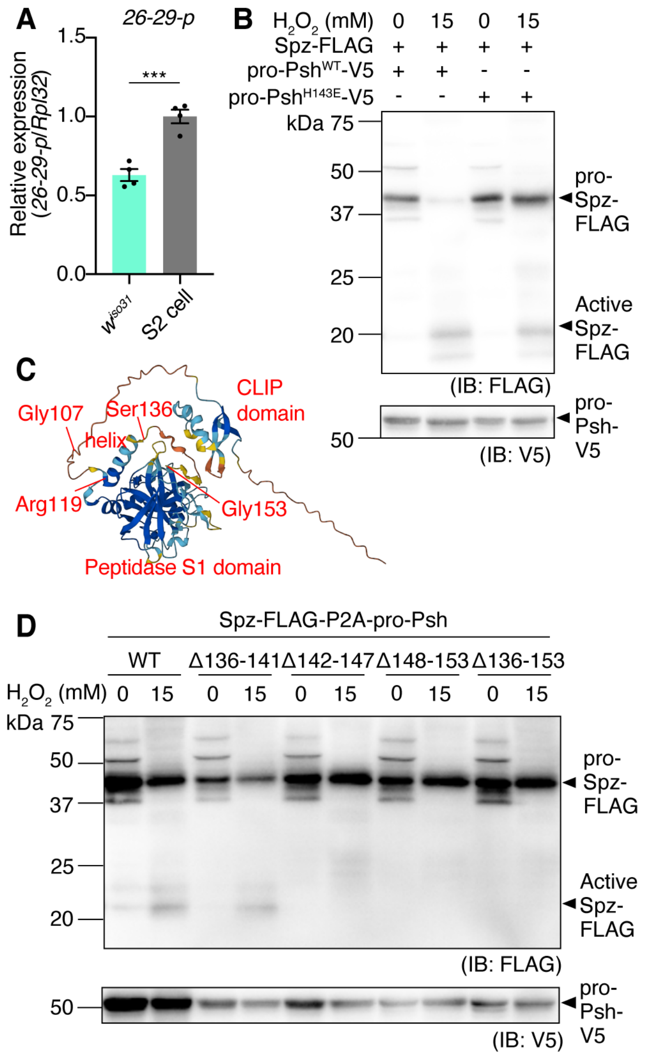

S10A–D Figs

2) Page 12, lane 249: the authors should justify why they do not consider a role for Easter

We appreciate your comment and agree with your suggestion. We additionally tested the expression levels of *SPE*, *MP1*, *easter*, *Hayan*, and *psh* in both S2 cell and whole-body fly samples (S4A–E Figs). The expression levels of *SPE*, *easter*, *Hayan*, and *psh* genes were markedly lower in S2 cells compared to those in whole-body fly samples (expression of *easter* in S2 cells is less than 1% of that in the fly samples). In contrast, the expression of *MP1* in S2 cells was comparable to that in the fly samples.

"While the expression levels of *SPE*, *easter*, *Hayan*, and *psh* genes were markedly lower in S2 cells compared to those in whole-body fly samples, that of the *MP1* gene was comparable (S4A–E Figs). Consistent with the transcript level, SPE protein was not detected in the S2 cell lysate (Fig 3D) (**lines 315–318**)"

"

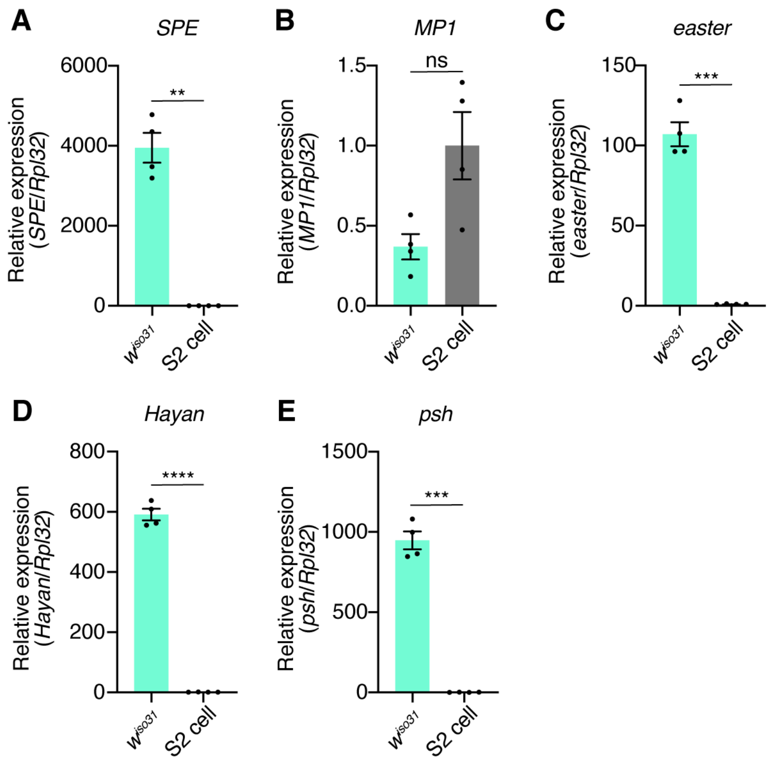

S4A–E Figs

Minor points

3) Page 10, lane 204: the authors may want to tone done the claim that the importance of Spz cleavage has not been investigated in vivo (e.g., PMID: 10489372, 12872120).

We appreciate and agree with your points, and have omitted the strong phrasing (**line 247–249**).

"Two SPs, Easter and SPE, cleave Spz during embryogenesis and infection, respectively (17,41)~~; however, it has not been investigated whether Spz cleavage is essential for Toll~~

~~activation in vivo~~. To test the requirement of Spz cleavage in apoptosis-deficient flies, **(lines 247–249)**"

4) Page 14, lane 290: please comment on the discrepancy between Fig5 A,B (no cleavage of Pro-Spz in the presence of Pro-Psh) and Fig S3B, and provide a more quantitative assessment than "strongly enhanced".

We appreciate your comment. We have noted in the main text (**lines**) that there are discrepancies between several figures regarding Spz cleavage in S2 cells in the presence of pro-Psh. We think that the variations in the amount of overexpressed pro-Psh between samples likely caused these differences in Spz cleavage. It is possible that when the amount of pro-Psh exceeds a certain threshold, Psh is auto-activated a small degree. We also assessed the enhancement of Spz cleavage by H_2O_2 treatment by calculating the ratio of signals of active Spz/pro-Spz (S7B Fig) (**lines 416–419 and 395–397**).

"Notably, in Fig 5A, Spz cleavage was not observed when only overexpressing pro-Psh, unlike the result seen in S7B Fig. This difference is potentially caused by variation in the amount of overexpressed pro-Psh between samples. It is possible that when the amount of pro-Psh exceeds a certain threshold, Psh is also auto-activated to a small degree. (**lines 416–419**)"

"A small amount of Spz was cleaved with the overexpression of pro-Psh, and this cleavage was strongly enhanced by approximately 4-fold upon treatment with H_2O_2 (S7B Fig). **(lines 397–399)**"

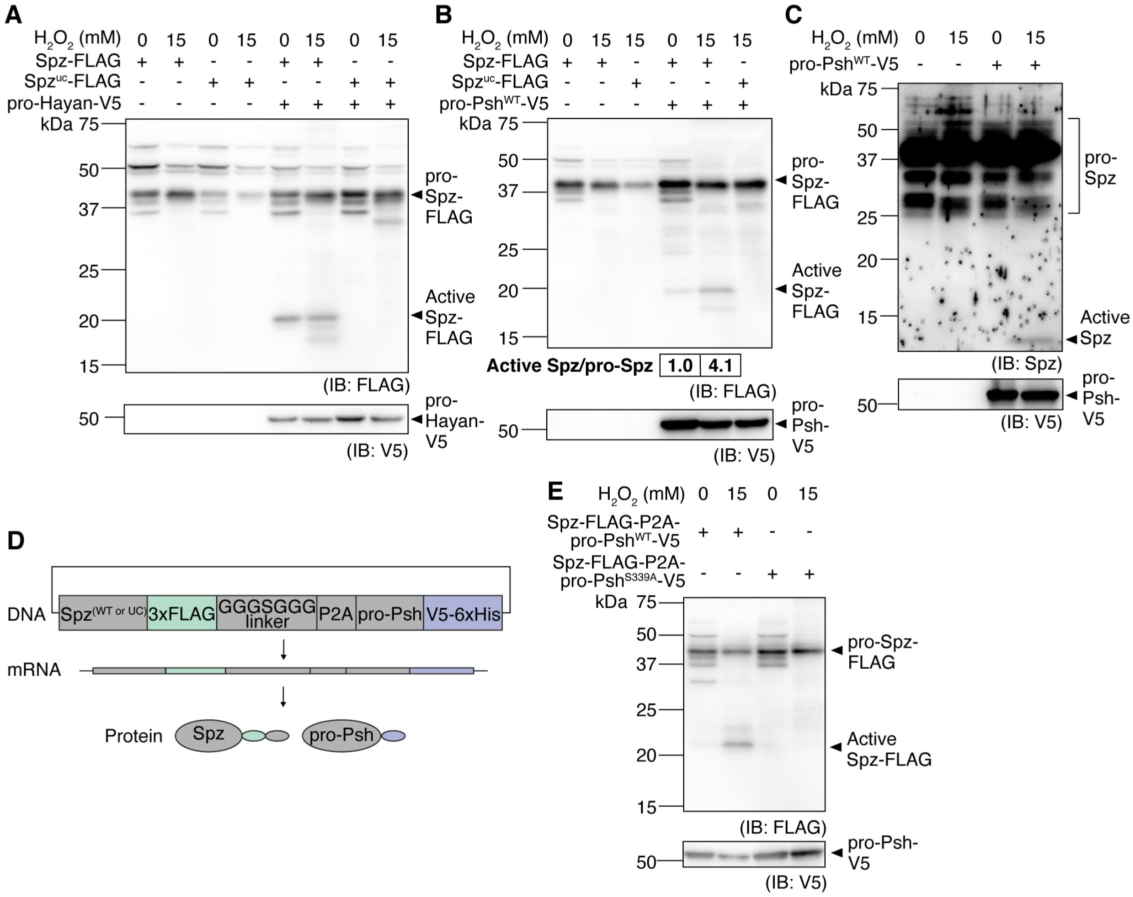

S7A–E Figs

Have all data underlying the figures and results presented in the manuscript been provided?

Large-scale datasets should be made available via a public repository as described in the *PLOS Genetics* data availability policy, and numerical data that underlies graphs or summary statistics should be provided in spreadsheet form as supporting information.

Reviewer #1: Yes

Reviewer #2: Yes

PLOS authors have the option to publish the peer review history of their article (what does this mean?). If published, this will include your full peer review and any attached files.

If you choose “no”, your identity will remain anonymous but your review may still be made public.

Do you want your identity to be public for this peer review? For information about this choice, including consent withdrawal, please see our Privacy Policy.

Reviewer #1: **Yes:** Bruno Lemaitre

Reviewer #2: No
